# Determinants and progression of stigma in amyotrophic lateral sclerosis/motor neuron disease

**DOI:** 10.1080/21678421.2024.2435969

**Published:** 2025-01-03

**Authors:** Carolyn A. Young, Amina Chaouch, Christopher J. Mcdermott, Ammar Al-Chalabi, Suresh K. Chhetri, Caroline Bidder, Elizabeth Edmonds, Cathy Ellis, Joe Annadale, Lisa Wilde, Basil Sharrack, Andrea Malaspina, Oliver Leach, Roger Mills, Alan Tennant

**Affiliations:** 1 Walton Centre NHS Foundation Trust, Liverpool, UK; 2 University of Liverpool, Liverpool, UK; 3 Greater Manchester Centre for Clinical Neurosciences, Salford, UK; 4 Sheffield Institute for Translational Neuroscience, Sheffield, UK; 5 Department of Basic and Clinical Neuroscience, Maurice Wohl Clinical Neuroscience Institute, King’s College London, London, UK; 6 Lancashire Teaching Hospital, Preston, UK; 7 Swansea Bay University Health Board, Port Talbot, UK; 8 North East London NHS Foundation Trust, Rainham, UK; 9 Dartford & Gravesham NHS Trust, Dartford, UK; 10 Hywel Dda University Health Board, Carmarthen, Wales, UK; 11 North West Anglia NHS Foundation Trust, Peterborough, UK; 12 United Lincolnshire Hospitals NHS Trust, Lincoln, UK; 13 UCL Queen Square Institute of Neurology, London, UK; 14 Royal Cornwall Hospitals NHS Trust, Truro, UK; 15 Leeds Institute of Rheumatic and Musculoskeletal Medicine, University of Leeds, Leeds, UK

**Keywords:** TONiC study, stigma, patient reported outcomes, Rasch analysis

## Abstract

*Objective*: Stigma in amyotrophic lateral sclerosis/motor neurone disease (ALS/MND) may be felt or enacted; felt stigma covers feeling devalued by the illness, whereas enacted stigma refers to being treated differently because of it. Stigma in ALS/MND has been shown to increase social withdrawal, worsen quality of life, and reduce use of assistive devices, so we explored prevalence and factors influencing stigma. *Methods*: Participants in the Trajectories of Outcome in Neurological Conditions-ALS study completed scales measuring stigma, fatigue, spasticity, functioning, mood, worry, self-esteem, and perceived health, as well as demographic information and symptoms like head drop or emotional lability. Following transformation to interval-scale estimates, data were analyzed by regression, structural equation modeling, and trajectory models. *Results*: Stigma was experienced by 83.5% of 1059 respondents. Worry, disease severity (King’s stage ≥ 3), emotional lability, fatigue, spasticity, and bulbar onset increase stigma. In contrast, increasing age, living with spouse/partner, and greater self-esteem were associated with reduced stigma. Trajectory analysis over 30 months (*N* = 1049) showed three groups, the largest (70.2%) had high levels of stigma which significantly increased during follow-up. In a recently diagnosed subset of 347 participants, stigma was experienced early in the disease course (<7 months after diagnosis), and for 77.2% stigma significantly increased over time. *Conclusions*: Both felt and enacted stigma are frequently perceived by people living with ALS/MND. Younger people and those with bulbar onset, emotional lability, worry, fatigue, and spasticity, or at more advanced clinical stages, are at greater risk.

## Introduction

People who manifest health problems are known to be at risk of stigma. Scambler described health-related stigma as “a social process, experienced or anticipated, characterized by exclusion, rejection, blame or devaluation that results from experience, perception or reasonable anticipation of an adverse social judgement about a person or group” (1). Stigma may be felt or enacted; felt stigma covers the shame of being different and fearing being treated badly, whereas enacted stigma refers to being discriminated against or excluded (2). In everyday life, felt and enacted stigma are intertwined: the person with the health condition fears being treated differently and any stigmatizing experience makes them more pessimistic about the next occasion. Stigma in amyotrophic lateral sclerosis/motor neurone disease (ALS/MND) has been shown to increase social withdrawal (3), and reduce quality of life (4) and use of assistive technology and devices (5).

A mixed methods study of people with ALS/MND (pwALS) found two manifestations of enacted stigma: social exclusion and stigmatizing behaviors displayed by others (e.g. staring), as well as three manifestations of felt stigma: alienation (e.g. embarrassment), perceived discrimination (e.g. feeling judged), and anticipated stigma (e.g. fear of exclusion) (6). More bulbar symptoms, higher King’s clinical stage, younger age, and living without a partner were significantly associated with enacted/felt stigma among pwALS. Another study in those with neurological disorders found spasticity was embarrassing and stigmatizing in social situations (7).

Some evidence exists that increasing self-esteem may offset stigma, facilitating continued employment as well as improved quality of life (8, 9). However, findings on the correlation between self-esteem and stigma appear conflicting (10, 11). Marital support appears to influence self-esteem over time (12).

The wide range of factors that might influence stigma in ALS/MND can be mapped to the well-known Wilson and Cleary model relating biological variables, symptoms, functioning, perceived health, and quality of life, with personal and psychological factors influencing the pathways (13). The potential determinants include clinical, demographic, symptoms, functioning, environmental, and psychological aspects, with particular consideration given to possible mediating effects. The current study reports on the influence of a range of these factors upon reported self-perceived stigma, whether such influences remain important over time, and how, if at all, stigma varies over time. It is derived from the Trajectories of Outcomes in Neurological Conditions-ALS (TONiC-ALS) study in the UK (14).

## Methods

### Main sample and data collection

Participants with ALS/MND were recruited into the TONiC-ALS study from specialist clinics and teams across the United Kingdom, data collected between 2013 and 2019 was used for this analysis to avoid influence of SARS-CoV-2 pandemic on stigma (15). Severity was graded using the King’s staging system (16). Following signed informed consent, all participants completing a baseline pack were eligible for follow-up with repeat packs at least 4 months apart, with an 11-month interval from baseline to first follow-up. Ethical approval was granted from local research committees (reference 11/NW/0743).

### Outcome measures

The questionnaire pack included patient reported outcome measures (PROMs), as well as indicators of various symptoms such as drooling and emotional lability. Based on the literature findings described in the introduction, the following PROMs were included in this analysis:

1. *Stigma:* The Stigma Scale for Chronic Illness (SSCI-8), measuring both felt and enacted stigma, where a higher score indicates more experienced stigma (17);
2. *Physical Function:* Amyotrophic Lateral Sclerosis Rating Scale revised (ALSFRS-R), where a higher score indicated better functioning, now with metric conversion (18, 19);
3. *Spasticity:* Spasticity Index-ALS, where a high score indicates more spasticity (20);
4. *Fatigue:* Neurological Fatigue Index-MND (NFI-MND), the 8-item summary scale scored 0–24, where higher scores represent greater fatigue (21);
5. *Breathlessness*: Dyspnea-12, scored 0–36 where a higher score indicates greater breathlessness, recently validated in ALS/MND (14, 22);
6. *Worry*: Penn State Worry Questionnaire, where a higher score indicates greater worry (23);
7. *Anxiety and Depression:* Hospital Anxiety and Depression Scale-depression subscales (HADS-D and HADS-A) where a higher score indicates greater symptomology (24);
8. *Self-Esteem:* Rosenberg Self-Esteem Scale (RSES) where a higher score indicates greater self-esteem (25);
9. *Self-Efficacy:* General Self-Efficacy scale, scored 0–30 where a higher score indicates higher-self efficacy (26);
10. *Perceived Health:* EQ-5D-5L and Visual Analog Scale (VAS), where higher scores indicate better health status and perceived-health respectively (27, 28).

Other indicators were included, consistent with the findings of the studies above:

1. Grading of disease severity: by King’s stage;
2. Marital status: as married/living with partner, or not;
3. Bulbar: bulbar onset or not;
4. Age;
5. Gender.

Participants completed a Symptom Inventory covering the following six symptoms; the subject was asked if they experienced the symptom and if so, whether it bothered them:

1. Muscle twitching;
2. Cramps;
3. Head drop;
4. Drooling;
5. Choking;
6. Emotional lability.

### Measurement and statistical analysis

All PROMs were transformed to interval scale equivalents by the Rasch model (29), using previously developed nomograms where available (14, 19–21, 30). Fit of data to the Rasch model for the Stigma Scale for Chronic Illness (SSCI-8), Penn State Worry, and Rosenberg Self-Esteem scales are reported in the Supplementary File. Symptoms were included as single indicator variables (none, experienced but not bothered by, bothered by). In the baseline cross-sectional sample, an exploratory regression analysis was used to identify variables that might influence stigma (i.e. significant predictors). This provided a selection of clinical factors, person factors, symptoms, and psychological factors, consistent with the Wilson and Cleary model (13), which were then examined using structural equation modeling (SEM). For the SEM, the sample size required varied by the model specification, given the number of observed and latent variables in the model. For example, with an anticipated effect size (0.2), desired probability (0.05), and statistical power levels (0.80), with two latent variables and six directly observed variables, a sample of 223 is required to both detect effect (i.e. the magnitude of influence of one variable upon another) and confirm model structure (31). The large cohort size in the current study allows for training and validation samples for cross-validation, but for an SEM with an effect size of 0.1, a sample size of 947 is required to confirm model structure, so the data from the full study were also used. Invariance was tested for gender. Magnitude of the overall effect is evaluated by the R^2^ upon stigma (32).

In the longitudinal sample, the probability of transition from one quartile group to another was examined, to assess whether stigma was fixed over time. Group-based trajectory models (GBTM) were used to identify trajectories, each representing an average path over time, for which stigma might reduce or increase, based upon the level of the intercept (33). The analysis was adjusted for differential group dropout rates (34).

Full details of the Rasch and interpretation, validity of the SEM, and requirements for identifying groups in the GBTM are given in the Supplementary File. All analyses were undertaken with STATA18 (35).

## Results

### Baseline data

A total of 1059 people with ALS/MND contributed to this analysis. With a mean age of 65.0 years (SD 10.5), mean duration since diagnosis was 23.5 months (SD 41.7); 60.7% were male. Most were married/living with partner (77.2%). Over half (55.2%) were at King’s stage 3 or above; 26.5% had bulbar onset (36).

Almost half (48.3%) reported some level of breathlessness. As expected, those few with respiratory onset had the highest level of breathlessness, significantly different to other onset types (Table 1). Over three-quarters (77.3%) reported some level of spasticity which also differed significantly across onset type. Almost all (97.8%) reported some level of fatigue. The mean health utility value was 0.595 (SD 0.264) and the mean perceived health VAS was 61.4 (SD 21.3). The mean of the ALSFRS-R (metric) was 25.0 (SD 5.7) or 33.2 (SD 8.3) on the ordinal.

**Table 1.** Demographic, clinical, symptoms, functioning, and psychological factors by onset type.

	Onset type						Significance	
Characteristics	Bulbar	Limb	Respiratory	Total	Test^a^	df	*p* ^a^	Range^b^
Demographic								
Age (years)	67.3	64.0	69.6	65.0	12.3	2/1056	<0.001	20–90
Gender (% male)	48.0	64.7	85.7	60.7	29.5	2	<0.001	
Married (%)	78.3	76.5	90.5	77.2	2.5	2	0.284	
Clinical								
Duration (months)	16.9	26.2	16.5	23.5	5.45	2/1051	0.004	0–554
King’s stage %								
0	0.0	0.9	0.0	0.66				
1	21.7	17.7	9.5	18.6				
2	18.5	28.5	9.5	25.5				
3	31.7	37.1	9.5	35.1				
4a	13.9	1.3	0.0	4.6				
4b	14.2	14.4	71.4	15.5				
Total	26.5	71.5	2.0	100.0	135.6	10	<0.001	
Symptoms								
Breathlessness	6.0	4.5	16.3	5.2	30.3	2/1056	<0.001	0–36
Spasticity	16.8	20.8	11.2	19.6	14.9	2/1056	<0.001	0–60
Fatigue	11.5	13.2	13.9	12.8	12.4	2/1056	<0.001	0–24
Functioning and health status								
Functioning	24.3	25.3	21.5	25.0	7.0	2/1056	0.001	0–48
Health status	0.708	0.556	0.530	0.595	36.2	2/1045	<0.001	−0.28–1.0
Perceived health	65.3	60.1	54.4	61.4	7.1	2/1034	0.001	0–100
Psychological								
Self-Efficacy	16.9	16.5	16.6	16.6	0.3	2/1033	0.757	0–30
Self-Esteem	16.6	16.1	16.4	16.2	1.0	2/1038	0.361	0–30
Stigma	10.2	8.6	8.6	9.0	9.6	2/1042	<0.001	0–32
*N*	281	757	21	1059				

df: degrees of freedom.

^a^Chi-square or ANOVA as appropriate.

^b^For continuous variables and full operational range of patient reported outcome measure.

Over four in five (83.5%) reported some level of stigma, 67.8% reported one or more aspects at the level of “sometimes” and above, and 28.8% at the level of “often” or “always.” The frequency of those reporting both an aggregated “sometimes, often, always” response and an aggregated “often, always” response for each item in the SSCI-8 is shown in Table 2. The most commonly reported item was “I felt embarrassed by my physical limitations,” while the least was “Some people acted as though it was my fault I have this illness.” The affirmed items represent a mix of enacted and felt stigma.

**Table 2.** Frequency of aggregated responses “sometimes, often, always” (SOA) and “often, always” (OA), for each item in the SSCI-8.

Item	SOA %	OA %
I felt embarrassed because of my physical limitations.	46.1	16.4
Because of my illness, I felt left out of things.	41.6	13.4
I felt embarrassed about my illness.	36.7	12.7
Because of my illness, some people seemed uncomfortable with me.	26.5	5.6
Because of my illness, some people avoided me.	24.6	5.6
Because of my illness, people avoided looking at me.	13.9	2.4
Because of my illness, people were unkind to me.	3.8	1.3
Some people acted as though it was my fault I have this illness.	3.3	0.6

Ordered by frequency.

The mean level of stigma was 9.0 (SD 5.3). Overall, females reported higher levels of stigma than males (*t* = 2.765, df 1043; *p* = 0.005), and there was a strong gradient by age, with those aged under 55 years reported significantly higher levels than those aged 65 years and older (*F* 15.4, df 3, 1041; *p* < 0.001). Those with bulbar onset also reported higher levels, compared to limb and respiratory (*F* 9.6; df 2, 1042; *p* ≤ 0.001). Those at King’s stage 3 and above also reported higher levels of stigma (*t* = 8.3, df 1043; *p* ≤ 0.001).

### Exploratory regression

Initially, 34 variables were included in an exploratory regression with stigma as the dependent variable. These included clinical variables, symptoms, functioning, and psychological variables. Finally, age, duration, bulbar onset, drooling, emotional lability, worry, self-esteem, and spasticity were identified as potential predictors and included in a final set (Table 3).

**Table 3.** Exploratory linear regression: final set.

Dependent stigma	Coef.	St. err.	*t* Value	*p* Value	[95% conf. interval]		Sig.	
Demographics								
Age	−0.095	0.012	−7.66	0.000	−0.120		−0.071	***
Clinical								
Duration (months)	0.018	0.003	5.38	0.000	0.011		0.024	***
Bulbar onset	1.898	0.344	5.52	0.000	1.223		2.573	***
Symptoms								
Fatigue	0.071	0.031	2.29	0.022	0.010	0.132		**
Spasticity	0.041	0.012	3.49	0.001	0.018	0.063		***
Drooling	0.688	0.195	3.53	0.000	0.305	1.07		***
Emotional Lability	0.323	0.171	1.89	0.059	−0.012	0.659		*
Perceived health								
Perceived health (VAS)	−0.046	0.007	−6.38	0.000	−0.060	−0.032		***
Psychological								
Self-Esteem	−0.213	0.030	−7.09	0.000	−0.272	−0.154		***
Worry	0.082	0.012	6.73	0.000	0.058	0.106		***
Constant	16.085	1.292	12.45	0.000	13.549	18.62		***
*R*-squared	0.420							
*F*-test	71.501		Prob. > *F* 0.000					

Coef.: coefficient; St. err.: standard error; Conf.: confidence; Sig.: significance; Prob.: probability.

***p < 0.01, **p < 0.05, *p < 0.1.

### Structural equation models

**Figure 1.**
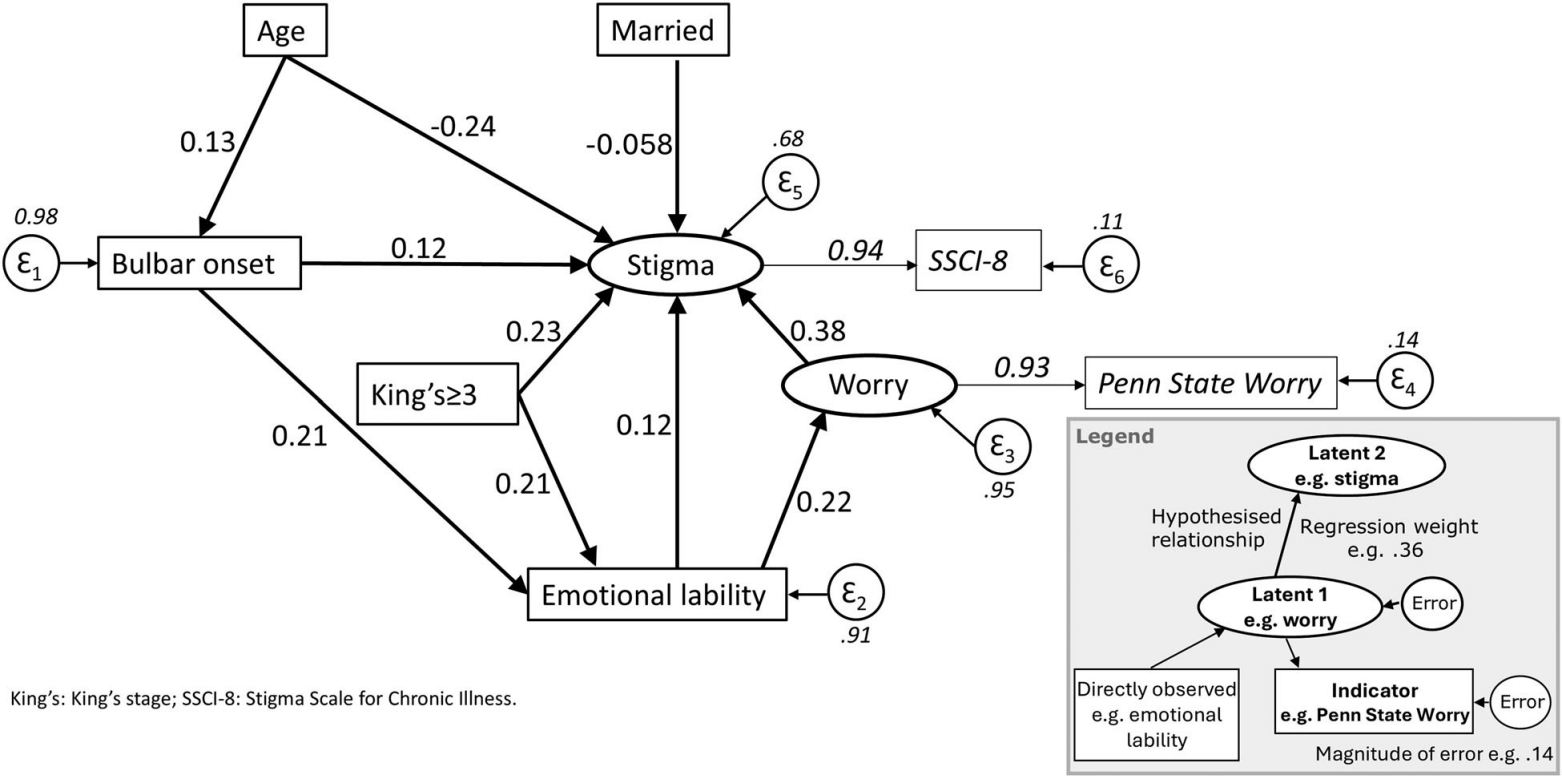
Model 1: Exploratory regression and literature guided model—Full sample.

*Model 1*: this was guided by exploratory regression results identifying factors associated with stigma, as well as factors identified in the literature. The data were randomized into two equal samples, “training” and “validation.” Fit was good in the training sample after some adjustment and simplification indicated by the modification indices (Table 4). The resulting model (Figure 1) was fully validated (Table 4: Model 1). The full sample also showed adequate fit. All paths were significant and invariant for gender (structural and measurement coefficients; score test). Examination of the total effects of the variables in the model using the full sample showed that worry was the strongest influence upon stigma, followed by King’s stage 3 or above, emotional lability, and bulbar onset (Table 5). In contrast, increasing age and living with spouse or partner were both associated with a reduced level of stigma.

**Table 4.** Fit of SEM models.

Model	Sample	Chi-square			RMSEA			CFI	TLI	*R* ^2^	*N*
		Value	df	*p*	Value	LCI	UCI				
1	Training	7.06	8	0.530	0.000	0.00	0.047	1.000	1.000	0.34	538
	Validation	9.32	8	0.315	0.018	0.00	0.056	0.994	0.987	0.29	550
	Total	13.0	8	0.112	0.024	0.00	0.047	0.990	0.977	0.32	1118
2	Training	20.56	9	0.015	0.049	0.21	0.077	0.984	0.949	0.45	538
	Validation	9.58	9	0.385	0.011	0.00	0.051	0.999	0.997	0.42	521
	Total	23.02	9	0.006	0.038	0.19	0.058	0.990	0.969	0.43	1059

SEM: structural equation modeling; RMSEA: root mean squared error of approximation; CFI: comparative fit index; TLI: Tucker–Lewis index; *R*^2^: coefficient of determination; df: degrees of freedom; LCI: lower confidence interval; UCI: upper confidence interval.

**Table 5.** Standardized effects of variables upon stigma.

Variable	Direct	Indirect	Total	*R* ^2^
Model 1-full sample				0.32
Increasing stigma				
Worry	0.381	–	0.381	
King’s stage ≥ 3	0.229	0.043	0.272	
Emotional lability	0.119	0.083	0.203	
Bulbar onset	0.118	0.043	0.161	
Decreasing stigma				
Increasing age	−0.244	0.021	−0.223	
Married/Partner	−0.058	–	−0.058	
Model 2-full sample				0.43
Increasing stigma				
Fatigue	0.122	0.233	0.355	
Spasticity	0.108	0.204	0.312	
Worry	0.146	0.148	0.294	
Bulbar onset	0.192	−0.030	0.162	
Emotional lability	0.092	0.052	0.144	
Decreasing stigma				
Self-Esteem	−0.357	–	−0.357	
Increasing age	−0.189	−0.039	−0.238	

Both structural and measurement coefficients were invariant to gender (Score test). Subject to rounding error.

**Figure 2.**
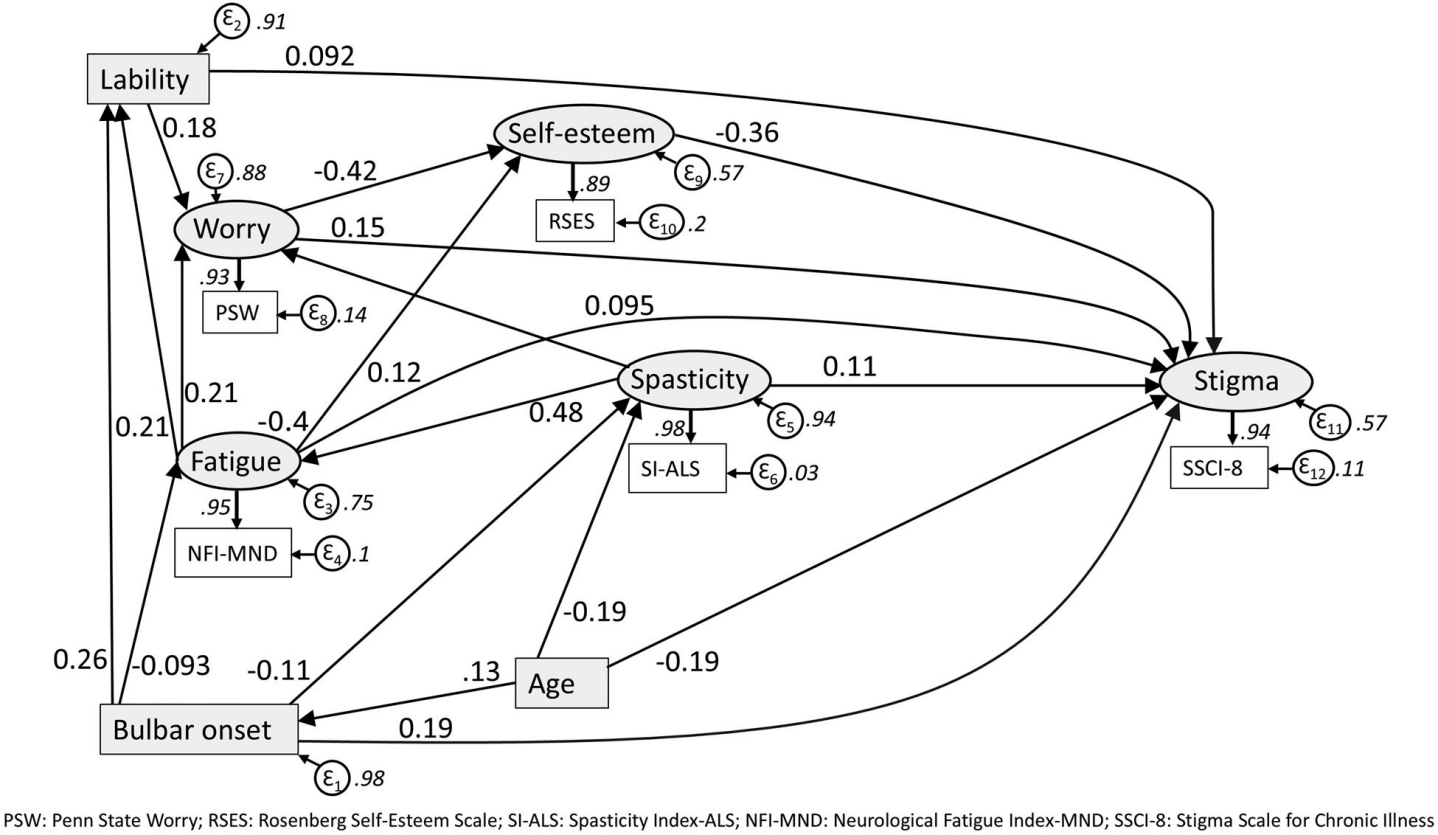
Conceptually driven model: Validation sample.

*Model 2*: this was a conceptually-based model with clinical, demographic, symptoms, and psychological variables (Figure 2). Fit in the training, validation, and total samples was adequate, although slightly weaker in the full data (Table 4: Model 2). Fatigue, spasticity, and worry showed the highest standardized effects increasing stigma (Table 5: Model 2). Higher self-esteem and increasing age reduced stigma. Of interest, fatigue is partially mediated by both self-esteem and worry, while the worry is itself mediated by self-esteem. It should also be noted that all variables other than self-esteem displayed both direct and indirect effects, indicating that each worked behind the scenes as well as directly.

### Longitudinal analysis

After an average time of 11.6 months, 397 (37.5%) entered the follow-up study (Table 6a), with no significant differences between those followed-up or not for age, duration or onset type (Table 6b). However, there was a significant difference by King’s stage ≥ 3 (45.6% compared with 54.4% King’s stage < 3; Chi-Square 23.9, df1, *p* ≤ 0.001).

**Table 6a.** Numbers of participants and time intervals in longitudinal study.

	Number of subjects	Time interval since last completion
Baseline	1059	–
Follow-up 1	397	11.6^a^
Follow-up 2	222	7.2
Follow-up 3	122^b^	6.7
Follow-up 4	62^b^	5.3

^a^Time to obtain ethical approval across over 30 sites.

^b^Influenced by hard stop related to COVID-19 pandemic.

**Table 6b.** Comparison between participants who did or did not enter the longitudinal study.

	*t* Value	Chi-square	df	*p* Value
Age at baseline	1.31		1057	0.188
Duration since diagnosis	0.232		1058	0.817
Onset type		3.157	2	0.206

df: degrees of freedom.

The transitional probabilities of the quartile distribution of stigma from baseline to first follow-up, expressed as percentages, are shown in Table 7. The key finding here is that stigma is not static, neither does it just increase. Rather, for those in the inter-quartile range of stigma, stigma both increases and decreases. Overall, 52.2% remained stable, 29.9% experienced increased stigma, while for 17.9% stigma decreased. However, 25% of the changes between baseline and first follow-up, for those within the interquartile range at baseline, were within the error of the scale.

**Table 7.** Transitional probabilities (%) within quartile-based stigma groups.

Stigma	Stigma follow-up				Total
	0	1	2	3	
0	**58.1**	22.9	8.6	10.4	100.00
1	16.9	**39.5**	33.1	10.5	100.00
2	4.1	28.8	**41.1**	26.0	100.00
3	2.3	6.7	19.1	**71.9**	100.00
Total	22.3	25.6	24.8	27.4	100.00

Bold represents stability between baseline and follow-up.

**Figure 3.**
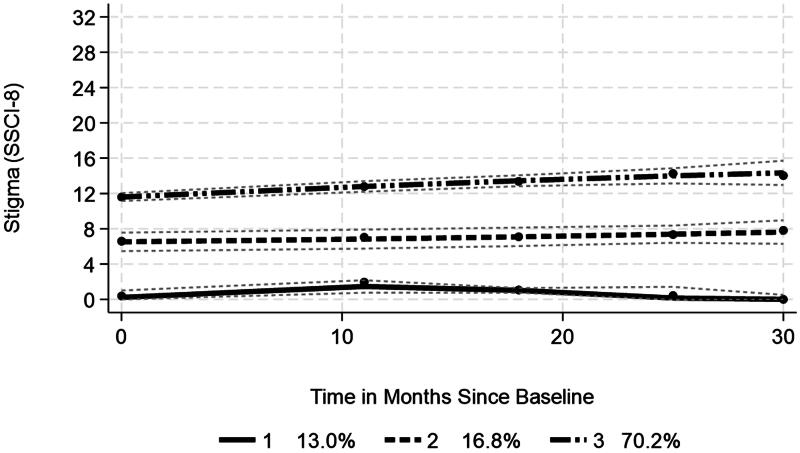
Trajectories of stigma over time—Full sample (*N* = 1049).

Trajectory analysis (*N* = 1049) showed three groups with probability of group assignment >0.8, essentially determined by the difference in the baseline level of stigma (Figure 3). Group 1 (13.0% of total group) showed low levels of stigma with a significant nonlinear trend, rising and then falling. Group 2 (16.8%) had higher levels of stigma, which were stable over time, while the largest group 3 (70.2%) had the highest levels of stigma at study entry and furthermore showed a significant increase in stigma over 30 months.

**Figure 4.**
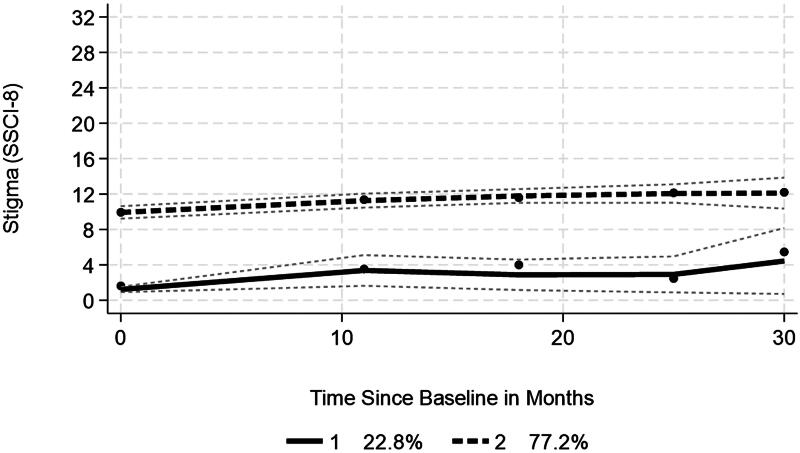
Trajectories of stigma over time—Inception group (*N* = 347).

A similar pattern was also observed for those in an inception group with duration since diagnosis <7 months upon entry to the study (*N* = 347) (Figure 4). Here two groups were identified with high probabilities (>0.93) of assignment to the groups, with one group (22.8%) showing a low but non-linear trajectory, and the second (77.2%) showing a higher level at entry to the study, with a significant increase. The difference across groups were similar to the full sample. In group 2, higher perceived health was assorted with lower stigma, while greater worry was associated with higher stigma. The point here is that stigma is manifest soon after diagnosis and the majority appear to follow a similar increasing pattern over time.

A multinomial logistic regression sought to characterize what, if anything, differentiated between the groups, using group 1 as the referent (comparison) group (Table 8). Compared to group 1, group 2 showed a lower risk of being in that group as age increased, as well as when perceived health increased. In contrast, the group displayed a higher risk as duration and worry increased. Group 3 showed that increasing age, better perceived health, and higher self-esteem reduced the risk of being in that group, compared to group 1, while bulbar onset (relative risk 3.6), longer duration, drooling, spasticity, and increased worry increased that risk. In other words, group 3 compared to group 1 were likely to be younger, with poorer perceived health and lower self-esteem, to have bulbar onset, longer disease duration, drooling, and spasticity.

**Table 8.** Multinomial logistic regression of trajectory groups—Full Sample.

Dependent stigma	RRR	St. err.	*t* Value	*p* Value	[95% conf. interval]		Sig.
Base Group 1							
Group 2							
*Demographic*							
Age	0.965	0.013	−2.65	0.008	0.940	0.991	***
Clinical							
Bulbar onset	1.171	0.441	0.42	0.675	0.560	2.448	
Duration (months)	1.011	0.004	2.54	0.011	1.003	1.02	**
*Symptoms*							
Drooling	1.098	0.254	0.40	0.687	0.697	1.729	
Fatigue	1.008	0.029	0.28	0.781	0.953	1.067	
Spasticity	1.011	0.011	0.95	0.341	0.989	1.033	
Perceived health	.980	0.008	−2.61	0.009	0.965	0.995	***
Psychological							
Emotional lability	1.11	0.233	0.50	0.619	0.736	1.676	
Worry	1.042	0.013	3.24	0.001	1.016	1.068	***
Self-Esteem	0.992	0.027	−0.31	0.755	0.940	1.046	
Constant	15.974	22.15	2.00	.046	1.055	241.933	**
Group 3							
Demographic							
* *Age	0.938	0.012	−5.11	0.000	0.916	0.962	***
Clinical							
* *Bulbar onset	3.563	1.177	3.85	0.000	1.865	6.809	***
* *Duration (months)	1.013	0.004	3.12	0.002	1.005	1.022	***
Symptoms							
* *Drooling	1.544	0.313	2.14	0.032	1.038	2.298	**
* *Fatigue	1.027	0.027	1.01	0.312	0.975	1.082	
* *Spasticity	1.020	0.010	1.95	0.051	1.000	1.041	*
Perceived health	0.963	0.007	−5.34	0.000	0.949	0.976	***
*Psychological*							
* *Emotional lability	1.359	0.253	1.64	0.100	0.943	1.959	
* *Worry	1.053	0.012	4.40	0.000	1.029	1.078	***
* *Self-Esteem	0.897	0.023	−4.20	0.000	0.853	0.944	***
Constant	1919.621	2473.8	5.87	0	153.554	23997.73	***
Cragg & Uhler’s *R*^2^	0.355						
Chi-square	339.308		Prob. > chi^2^	0.000			

RRR: relative risk ratio; St. err.: standard error; Conf.: confidence; Sig.: significance; Prob.: probability.

***p < 0.01, **p < 0.05, *p < 0.1.

## Discussion

Stigma is a common problem in ALS/MND, reported at some level by 83.5% of a large sample of 1059 people. One or more aspects of felt or enacted stigma at the level of ‘sometimes’ and above were experienced by 67.8%. Age, duration, bulbar onset, drooling, spasticity, emotional lability, worry, and self-esteem were all found to be associated with stigma. A simple SEM model based on these findings, along with certain variables from the literature, showed worry, disease stage (King’s stage 3 or more), emotional lability, and bulbar onset all influenced stigma in descending order of importance. In contrast, increasing age and living with spouse or partner both reduced the level of stigma. These findings are consistent with previous reports (6). However, the conceptually based SEM model suggested a much more complex picture, including not just clinical and demographic factors, but also symptoms and various psychological factors. Each of the psychological factors of self-esteem and worry acted as mediators to stigma, along with the symptom of emotional lability. Overall, including both direct and indirect effects upon stigma, self-esteem showed the greatest effect, reducing stigma as self-esteem increased. Fatigue, spasticity, and worry were the strongest influences for increasing stigma.

Longitudinal analysis indicated that there was some variability in stigma over a 30-month period. Trajectory analysis identified three groups. The dominant group, with just over 70% of cases, entered the study with the highest levels of stigma, and showed a significant average increase over the follow-up. A multinomial logistic regression highlighted that this dominant group, with the highest level of stigma at baseline, were disadvantaged in many respects, compared to the referent group (sustained low stigma), including being in poorer health, and with lower self-esteem. Repeating the analysis for the inception group showed a similar pattern, which indicates that higher levels of stigma was present in over two-thirds soon after diagnosis.

There are many clinical implications to this work. Stigma is common and is worsened by specific symptoms that may be treated, potentially ameliorating stigma. Emotional lability, spasticity, and bulbar impairment increase the risk of stigma, perhaps through behaviors such as uncontrolled laughing/crying, spasms, or impaired speech. Treatment of symptoms and education of patient, family, and the public could be beneficial. Younger patients with bulbar onset and without a partner are more vulnerable, especially as the disease progresses; they are more likely to experience stigma at increased and worsening levels. Cognitive behavioral interventions for self-esteem have been trialed in other conditions, with improvements in the Rosenberg Self-Esteem score, offering a possible method of reducing stigma given the protective effects of greater self-esteem (37). Health facilities should address stigma which they may be enacting (38).

There are limitations to the study. One study relating to Parkinson’s disease suggested that cultural differences may influence the level of stigma (39). The current study is limited to just one country and mostly Caucasian patients. A second limitation is that in the UK there is a public health service, where everyone is able to access free health care. Elsewhere, systems with access requiring payment may affect how people, including family members, perceive pwALS and consequently the level of enacted stigma.

These findings suggest that more needs to be done to uncover other influences that affect stigma. One recent scoping review to map the existing evidence on stigma associated with neurological disease in adult populations identified three main gaps (40). These were low attention to stigma related to neurological diseases other than epilepsy, limited cross-cultural comparisons of stigma related to neurological disease, and the absence of gender as a variable in the analysis of stigma-related outcomes. The current study addresses the shortfall in understanding stigma in ALS/MND, and included gender. In the regression analysis gender was not significant, and in the SEM, findings were invariant to gender. Cross-cultural comparisons have yet to be made. Future work could also examine enacted and felt stigma, including whether they have different implications.

In conclusion, both felt and enacted stigma are frequently perceived by people living with ALS/MND. Younger people and those with bulbar onset, emotional lability, fatigue, and spasticity or at more advanced clinical stages are at greater risk. Clinical interventions to treat emotional lability, reduce worry, or improve self-esteem might all be means to ameliorate stigma. Future work should explore these and other potential determinants of stigma.

## Supplementary Material

Supplementary File revised MND stigma.pdf

## Data Availability

Data supporting this study are not openly available due to reasons of sensitivity and are available from the corresponding author upon reasonable request. Data are located in controlled access data storage at Walton Centre NHS Trust.
