# Supplementary material for: Determinants and progression of stigma in amyotrophic lateral sclerosis/motor neuron disease
Source: Amyotroph Lateral Scler Frontotemporal Degener. 2025 Jan 3;26(3-4):192–202. doi: 10.1080/21678421.2024.2435969 (PMC12011026; doi:10.1080/21678421.2024.2435969)
Supplement: Supplementary File revised MND stigma.pdf [file IAFD_A_2435969_SM5146.pdf]

Supplementary File

This supplementary material is from:

Determinants and progression of stigma in Amyotrophic Lateral Sclerosis/Motor Neuron Disease

CA Young, A Chaouch, CJ McDermott, A Al-Chalabi, S Chhetri, C Bidder, E Edmonds, C Ellis, J Annadale, Lisa Wilde, B Sharrack, A Malaspina, O Leach, RJ Mills, A Tennant, On behalf of the TONiC-ALS study group

Corresponding author: Cayoung@liverpool.ac.uk; orcid id: 0000-0003-1745-7720

Contents

1.1. Why use Rasch Measurement Theory? 2
1.2. Methods of Rasch Analysis 2
Supplementary File: Table 1. Strategies seeking fit of the data to the model. 4
1.3. Structural Equation Modeling 5
1.4. Group-based trajectory model 5
1.5. Results of fit to the Rasch Model 6
1.5.1. SSCI-8 6
Supplementary File: Table 2. Nomogram for SSCI-8 in ALS/MND. Raw score to interval estimate 7
1.5.2. Penn State Worry 7
Supplementary File: Table 3. Nomogram for Penn State Worry scale in ALS/MND. Raw score to interval estimate. 9
1.5.3. Rosenberg Self Esteem 10
Supplementary File: Table 4. Nomogram for Rosenberg Self Esteem scale in ALS/MND. Raw score to interval estimate 10
2.0. Exploratory Regression 11
2.1. Correlation Matrix 11
Supplementary File: Table 5. Spearman's rank correlation coefficients. 11
2.2. Residual Analyses 12
2.2.1. Outliers 12
2.2.2. Residual Normality 12
2.2.3. Heteroscedasticity 12
2.2.4. Variance inflation factor 13
2.2.5. Ramsey RESET test for omitted variables 13
3.0. References 13

1.1. Why use Rasch Measurement Theory?

Using patient reported outcome measures (PROMs) for fundamental measurement requires two conditions: first that the measurement of person ability is independent of the distribution of items in the scale, and secondly that the calibration of item difficulty is independent of the distribution of the ability of persons in the sample. Out of all the models within the general Item Response Theory (IRT) framework, the Rasch model is unique because it is the only parametric model where the raw score over all items is a sufficient statistic for the person parameter. In clinical terms, if the PROM fits the Rasch model, the raw score arising from summing the individual item scores can generate a valid measure of the trait (e.g. stigma) in that person. Due to this property, conditional maximum likelihood (CML) estimation can be used to estimate item parameters consistently without assuming a specific population distribution for the latent trait. Furthermore, at the core of the Rasch model is the notion of homogeneity, i.e. the same ordering of items and persons irrespective of their level on the trait. So, for persons, regardless of where they are on the trait being measured, the ordering of items will always be the same, a requirement for fundamental measurement. This is not the case for other parametric IRT models, nor for factor analysis.

Finally, applying RMT can contribute to the latest developments in test equating, where scales measuring the same attribute are placed upon the same reference metric. This allows comparison across studies which use different scales for the same attribute, so facilitating meta-analysis [1].

1.2. Methods of Rasch Analysis

Data from each (sub)scale was tested against the requirements of the Rasch Measurement model [2]. Briefly, these requirements include: i) unidimensionality; ii) monotonicity; iii) homogeneity; iv) local independence; and v) group invariance [3, 4]. Whichever set of items are to be added together to provide a score, they should satisfy all of these requirements. They should: i) measure one thing (domain/ construct/trait); ii) the probability of a positive response to an item (or in the case of polytomous items, the transition from one response category to the next) should increase with underlying ability, as should the total score [5]; iii) the same hierarchical ordering of items should hold for each level (or grouping) of the score [6]; iv) items should

be conditionally (on the score) independent of one another [7]; and v) the response to items across different groups such as age or gender should, conditioned on the total score, be the same – referred to as (the absence of) Differential Item Functioning (DIF) [4].

Each requirement is tested. A t-test is used to determine if two separate groups of items deliver significantly different estimates, following the procedure given by Smith [8]. The hierarchical ordering of items across the scale is determined through a Chi-Square test of fit based on grouped scores. Monotonicity is evaluated through inspection of the item-category ordering. Conditional item dependence is determined though the correlation of residuals, where pair-wise correlations should not exceed 0.2 above the average residual [9]. Should clusters of locally dependent items be found, consideration is given to grouping these into 'super items' or testlets (simply adding them together to make one larger item, the latter based on *a priori* defined groups) to absorb the local dependency [10]. In the RUMM2030 software, this gives a bi-factor equivalent solution retaining a specified proportion of the variance. This "Explained Common Variance (ECV)" is reported, whereby a value less than 0.7 is indicative of requiring a multidimensional model, a value above 0.9 a unidimensional model, and the grey area in between, undetermined, requiring further evidence [11]. Consequently, value of the ECV at 0.9 and above is considered acceptable in the current analysis. If two parallel forms are created from either a subscale structure, if present, or from the pattern of local dependency in the item set, this requires a latent correlation ≥ 0.9. This is consistent with the reliability required for individual use [12]. Consequently, valid parallel forms would require both their latent correlation to be ≥0.9 and the ECV to be ≥0.9.

Group invariance (DIF) is tested through an ANOVA of residuals for factors such as age, gender, duration since diagnosis, education levels, and whether or not the patient is self-employed or employed, and working full-time or part-time. Should DIF be identified it is tested by a comparison of person estimates from split and unsplit solutions to see if it is 'substantive' [13]. Where the difference is significant (a paired t-test), the result is reported as an effect size where a value higher than 0.1 is considered to represent substantive DIF [14]. If this is present, then the scale works in different ways for the contextual factor under consideration, and results are reported separately. Finally reliability is reported as both a Person Separation Index (PSI), and as Cronbach's alpha. If data is normally distributed they are equivalent, but otherwise PSI tends to be lower the more data are skewed. Values are

treated the same, and so values below 0.7 would be described as low, as they do not support group use.

A hierarchical approach to seeking fit of the data to the model for existing scales is adopted, with level 1 as the priority (Supplementary file: Table 1). All aspects listed above must be met. Should a level 5 solution be unavailable, item deletion will be considered (level 6). If this fails, then level 7 will be utilised to test if the scale satisfies ordinal scaling; if not level 8 remains, indicating failure.

Supplementary File: Table 1. Strategies seeking fit of the data to the model.

Level	Nature	Adjustments	Reporting		
			Chi-Square	ECV ≥0.9	Latent Correlation ≥0.9
1	Item-based	None	Interaction	No	No
2	Item-Based	Clusters for Local Item Dependency	Interaction	Yes	No
3	Domain-based	On existing sub-scales >2	Interaction	Yes	No
4	Parallel Form	On existing sub-scales ≤2, or<br>2 local dependency patterns or conceptual groups	Conditional	Yes	Yes
5	Parallel Form	On alternative items	Conditional	Yes	Yes
6	Item Deletion	On all original items<br>Repeat Levels 1-5	Interaction	No	No
7	Mokken Scaling	On items if Unidimensional. Loevinger's coefficient H ≥0.4-moderate	No	No	No
8	Fail	No valid ordinal scale	No	No	No

ECV: Explained Common Variance

1.3. Structural Equation Modeling

Structural equation modeling (SEM) has been defined as various types of models to depict relationships among observed variables, with the same basic goal of providing a quantitative test of a theoretical model hypothesised by the researcher[15]. The theoretical model is almost always specified by a path diagram showing the hypothesised relationships between the various components of the model. These components may be directly observed variables, or latent variables. This reflects the approach used in the current study, a mix of both types of component with a special adaptation to include latent estimates derived from the Rasch analysis above. Generally latent variables should have at least three indicators, but here we employ single indicator latent variables [16]. Here, the error and regression parameters for the single indicator are pre-specified. The error is defined as the variance of the estimate multiplied by 1-apha, that is the reliability of the scale from which the estimates are derived. The regression weight is defined as the standard deviation multiplied by the square-root of alpha.

Given model specification, the task is to determine if the covariance matrix implied by the model is close enough to the sample covariance matrix so that the differences might reasonably be considered as being due to sampling error [17]. A number of fit statistics are available to test this. In the current study our primary indicator of fit is the χ^2, where a non-significant value would indicate that the model is consistent with the covariance data. In addition, a number of approximate fit indices are reported. These include the Root Mean Square Error of Approximation (RMSEA) where a value of the lower confidence interval < 0.05 would indicate a closely fitting model, and where a upper confidence interval > 0.08-0.10 would be considered unacceptable; the Comparative Fit index (CFI) and the Tucker Lewis Index (TLI) where both values should be above 0.95 [18]. If necessary, modification indices are used to improve model fit.

1.4. Group-based trajectory model

The time metric was the median month since the baseline questionnaire at each follow-up. Each domain was assessed at baseline and up to three further follow-ups, and modelled with a censored normal distribution. The number and shape (via polynomial functions) of trajectories were determined by analysing one to five group models without covariates. To

accommodate attrition, a 'dropout' model was applied, specified in its basic form of constant dropout across assessment occasions [19]. The Bayesian Information Criterion (BIC) was used to determine the best-fitting model, also with consideration for a useful and parsimonious model. Average posterior probabilities above 0.7 were also deemed to indicate optimal fit [20]. Missing data were handled using a maximum likelihood approach based on a missing-at-random assumption.

1.5. Results of fit to the Rasch Model

1.5.1. SSCI-8 [21]

The item set had not previously been fit to the Rasch model. considerable local item dependency was found in the scale. For example, with an average residual correlation of −0.12, the items "Because of my illness some people avoided me" (item 1) and the item "Because of my illness, people avoided looking at me" (item 3) had a residual correlation of 0.240. The locally dependent items were largely found in the upper part of the scale and so a two super item version was created based on the pattern of residuals.

The scale was found to have adequate fit to the model with an item and person standard deviation of 1.016 and 0.782 respectively. All items had ordered thresholds. Conditional Chi-Square test of fit based upon two super-items gave a χ^2 of 20.6 (df 20); p=0.420) with a reliability of 0.87. 97% of the variance was retained in this bi-factor solution. The unidimensionality test revealed just 1.5% of estimates differing between the two super-items. DIF was absent for age, gender, duration and time (of assessment). There was some variation by onset type but it was not possible from the ICC curves to distinguish which type varied. The transformation table for raw score to interval level is below (Supplementary file: Table 2).

Supplementary File: Table 2. Nomogram for SSCI-8 in ALS/MND. Raw score to interval estimate.

Raw Score	Interval Estimate
0	0.0
1	3.4
2	5.6
3	7.1
4	8.2
5	9.1
6	9.9
7	10.6
8	11.2
9	11.8
10	12.4
11	12.9
12	13.5
13	14.1
14	14.7
15	15.4
16	16.0
17	16.6
18	17.3
19	18.0
20	18.6
21	19.3
22	19.9
23	20.5
24	21.1
25	21.7
26	22.3
27	23.0
28	23.8
29	24.7
30	26.1
31	28.3
32	32.0

1.5.2. Penn State Worry [22]

The item set had not previously been fit to the Rasch model. Two of the 16 items had disordered thresholds. Local item dependency was observed throughout the scale. For example, the items “Once I start worrying I cannot stop” (item 14) and “I worry all the time” (item 15) had a residual correlation of 0.340. where the average residual correlation was

−0.05. A two testlet solution, based upon the pattern of dependency, that is the first eight and second eight items, showed adequate fit. Item and person standard deviations were 0.2695 and 1.0851 respectively. Conditional Chi-Square test of fit gave a χ^2 of 60.7 (df 56), and a significance level of 0.305. The scale had a reliability (α) of 0.94 and 99% of the variance was retained in the bi-factor solution.

Unidimensionality was confirmed with a t-test value of 3.8%. No DIF was found for any contextual factor, including time. The transformation table for raw score to interval level estimates is shown below (Supplementary file: Table 3).

Supplementary File: Table 3. Nomogram for Penn State Worry scale in ALS/MND. Raw score to interval estimate.

Raw score	Metric	Raw score (cont)	Metric (cont)
0	0.0	33	33.2
1	5.2	34	33.8
2	8.4	35	34.4
3	10.5	36	35.1
4	12.0	37	35.7
5	13.3	38	36.4
6	14.3	39	37.0
7	15.3	40	37.6
8	16.2	41	38.3
9	17.0	42	38.9
10	17.8	43	39.6
11	18.5	44	40.2
12	19.2	45	40.8
13	20.0	46	41.5
14	20.7	47	42.1
15	21.3	48	42.8
16	22.0	49	43.4
17	22.7	50	44.1
18	23.4	51	44.7
19	24.1	52	45.4
20	24.7	53	46.1
21	25.4	54	46.8
22	26.1	55	47.5
23	26.7	56	48.3
24	27.4	57	49.1
25	28.0	58	50.0
26	28.7	59	51.0
27	29.3	60	52.2
28	30.0	61	53.7
29	30.6	62	55.7
30	31.2	63	58.9
31	31.9	64	64.0
32	32.5		

1.5.3. Rosenberg Self Esteem [23]

The item set had not previously been fit to the Rasch model. All 10 items had ordered thresholds, however local item dependency was observed. For example, the items “At times I feel no good at all” and “I certainly feel useless at times” displayed a residual item correlation 0.176, where the average residual correlation was -0.11. It was noted that the pattern of residual correlations reflected the items which were swopped (i.e. positive items change to negative), and thus a two testlet solution was implemented, based upon the pattern of negative and positive worded items. Item and person standard deviations were 1.247 and 0.977 respectively. Conditional Chi-Square test of fit gave a χ^2 of 30.9 (df 22), and a significance level of 0.10. The scale had a reliability (α) of 0.86 and 94% of the variance was retained in the bi-factor solution. Unidimensionality was confirmed with a t-test value of 5.9% (LCI 4.4%). Some DIF was present but could not be differentiated by the ICC curves of the respective groups (e.g. onset). no DIF was observed for age, gender and time. The transformation table for raw score to interval level estimates follows (Supplementary file: Table 4).

Supplementary File: Table 4. Nomogram for Rosenberg Self Esteem scale in ALS/MND. Raw score to interval estimate.

Raw score	Metric	Raw score (cont)	Metric (cont)
0	0.0	16	18.9
1	3.0	17	19.8
2	5.1	18	20.6
3	6.6	19	21.3
4	7.7	20	22.0
5	8.7	21	22.6
6	9.6	22	23.1
7	10.5	23	23.7
8	11.4	24	24.2
9	12.2	25	24.8
10	13.1	26	25.4
11	14.1	27	26.1
12	15.0	28	27.0
13	16.0	29	28.2
14	17.0	30	30.0
15	18.0		

2.0. Exploratory Regression

2.1. Correlation Matrix

Supplementary File: Table 5. Spearman's rank correlation coefficients.

Variables	(1)	(2)	(3)	(4)	(5)	(6)	(7)	(8)	(9)	(10)	(11)
(1) Age	1.000										
(2) Duration (months)	-0.024	1.000									
(3) Bulbar onset	0.111	-0.078	1.000								
(4) Spasticity	-0.192	0.060	-0.119	1.000							
(5) Drooling	0.140	0.056	0.499	0.038	1.000						
(6) Fatigue	-0.104	0.077	-0.128	0.483	0.075	1.000					
(7) Perceived Health	0.020	-0.165	0.103	-0.306	-0.084	-0.504	1.000				
(8) Self-Esteem	0.012	-0.090	0.051	-0.307	-0.098	-0.446	0.432	1.000			
(9) Emotional lability	-0.042	0.072	0.218	0.131	0.343	0.170	-0.120	-0.211	1.000		
(10) Worry	-0.023	0.000	0.045	0.226	0.054	0.266	-0.181	-0.451	0.197	1.000	
(11) Stigma	-0.194	0.186	0.137	0.332	0.236	0.378	-0.376	-0.472	0.297	0.382	1.000

2.2. Residual Analyses

2.2.1. Outliers

	Low	High
# Mild outliers	6	1
% Mild outliers	0.58%	0.10%
# Severe outliers	0	0
% Severe outliers	0%	0%

2.2.2. Residual Normality

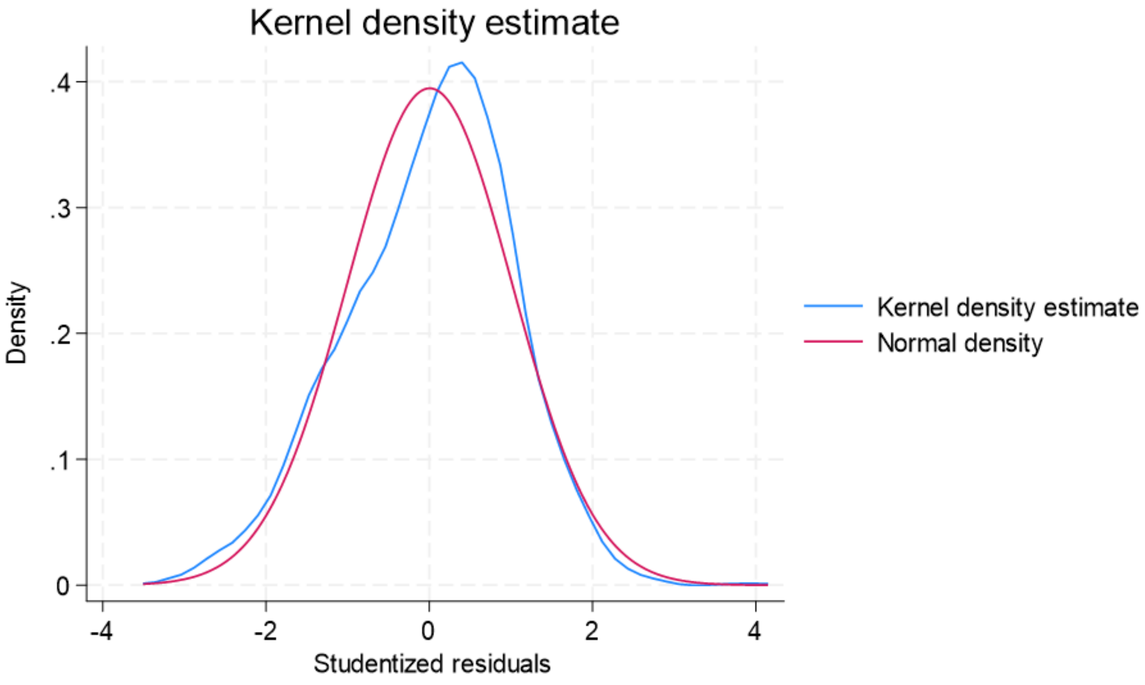

2.2.3. Heteroscedasticity

Breusch–Pagan/Cook–Weisberg test:

$chi^2(1) = 1.92$

$Prob > chi^2 = 0.1657$

2.2.4. Variance inflation factor

	VIF	1/VIF
NFI-MND	1.701	.588
RSES	1.614	.62
Drooling	1.542	.649
EQ5D-VAS	1.448	.69
Bulbar	1.438	.695
Spasticity	1.341	.746
PSW	1.306	.766
Emotional lability	1.243	.805
Age	1.077	.929
Duration	1.028	.973
Mean VIF	1.374	.

VIF: variance inflation factor; NFI-MND: Neurological Fatigue Index-MND; RSES: Rosenberg Self-Esteem Scale; VAS: visual analogue scale; PSW: Penn State Worry

2.2.5. Ramsey RESET test for omitted variables

F(3, 986) = 0.35 Prob > F = 0.7911

3.0. References

1. Prodinger B, Coenen M, Hammond A, Küçükdeveci AA, Tennant A. Scale Banking for Patient-Reported Outcome Measures That Measure Functioning in Rheumatoid Arthritis: A Daily Activities Metric. Arthritis Care Res (Hoboken). 2022;74(4):579-87.
2. Rasch G. Probabilistic Models for Some Intelligence and Attainment Tests. Chicago: The University of Chicago Press; 1980.
3. Gustafsson J. Testing and obtaining fit of data to the Rasch model. British Journal of Mathematical & Statistical Psychology. 1980;33(2):205-33.
4. Teresi JA, Kleinman M, Ocepek-Welikson K. Modern psychometric methods for detection of differential item functioning: application to cognitive assessment measures. Stat Med. 2000;19(11-12):1651-83.
5. Kang HA, Su YH, Chang HH. A note on monotonicity of item response functions for ordered polytomous item response theory models. The British journal of mathematical and statistical psychology. 2018;71(3):523-35.
6. Rost J. An unconditional likelihood ratio for testing item homogeneity in the Rasch model. Education Research and Perspectives. 1982;9(June):7-17.
7. Wilson M. Detecting and Interpreting Local Item Dependence Using a Family of Rasch Models. Applied psychological measurement. 1988;12(4):353-64.

8. Smith E. Detecting and evaluating the impact of multidimensionality using item fit statistics and principal component analysis of residuals. J Appl Meas. 2002;3:205-31.
9. Christensen KB, Makransky G, Horton M. Critical values for Yen's Q3: Identification of local dependence in the Rasch model using residual correlations. Applied psychological measurement. 2017;41(3):178-94.
10. Wainer H, Kiely G. Item clusters and computer adaptive testing: A case for testlets. J Educ Meas. 1987;24(3):185-202.
11. Quinn H. Bifactor Models, Explained Common Variance (ECV), and the Usefulness of Scores from Unidimensional Item Response Theory Analyses [Masters Thesis]. North Carolina: University of North Carolina at Chapel Hill; 2014.
12. Bland JM, Altman DG. Statistics notes: Cronbach's alpha. British Medical Journal. 1997;314:572.
13. Hagquist C, Andrich D. Recent advances in analysis of differential item functioning in health research using the Rasch model. Health Qual Life Outcomes. 2017;15(1):181.
14. Rouquette A, Hardouin JB, Vanhaesebrouck A, Sébille V, Coste J. Differential Item Functioning (DIF) in composite health measurement scale: Recommendations for characterizing DIF with meaningful consequences within the Rasch model framework. PLoS One. 2019;14(4):e0215073.
15. Schumacker RE, Lomax RG. A beginner's guide to structural equation modeling. 3rd edition ed. New York: Routledge; 2010.
16. Hayduk LA, Littvay L. Should researchers use single indicators, best indicators, or multiple indicators in structural equation models? BMC Med Res Methodol. 2012;12:159.
17. Kline R. Principles and Practice of Structural Equation Modeling. Third Edition ed. New York, London: Guilford Press; 2011.
18. West SG, Taylor AB, Wu W. Model fit and model selection in structural equation modeling. In: Hoyle RH, editor. Handbook of structural equation modeling: The Guilford Press; 2012. p. 209-31.
19. Haviland AM, Jones BL, Nagin DS. Group-based trajectory modeling extended to account for nonrandom participant attrition. Sociological Methods & Research. 2011;40(2):367-90.
20. Nagin DS, Odgers CL. Group-based trajectory modeling in clinical research. Annual review of clinical psychology. 2010;6:109-38.
21. Yamile Molina, Choi SW, Cella D, Rao D. The Stigma Scale for Chronic Illnesses 8-Item Version (SSCI-8): Development, Validation and Use Across Neurological Conditions. Int J Behav Med. 2013;20:450-60.
22. Meyer TJ, Miller ML, Metzger RL, Borkovec TD. Development and validation of the Penn State worry questionnaire. Behaviour Research and Therapy. 1990;28(6):487-95.
23. Rosenberg M. Society and the adolescent self image. Princeton, NJ: Princeton University Press; 1965.
